# Visual attention in 5-year-olds from three different cultures

**DOI:** 10.1371/journal.pone.0200239

**Published:** 2018-07-16

**Authors:** Moritz Köster, Shoji Itakura, Relindis Yovsi, Joscha Kärtner

**Affiliations:** 1 Department of Psychology, University of Münster, Fliednerstraße 21, Münster, Germany; 2 Institute of Psychology, Free University Berlin, Habelschwerdter Allee 45, Berlin, Germany; 3 Department of Psychology, Graduate School of Letters, Kyoto University, Yoshida-honmachi, Sakyo-ku, Kyoto, Japan; 4 Independent ECD consultant, Brussels, Belgium; University of Portsmouth, UNITED KINGDOM

## Abstract

Cognitive processes differ markedly between children from different cultures, with best evidence for attention to visual scenes and the activities of others. Children from urban Western cultures tend to focus on focal objects, whereas children from urban East-Asian cultures rather attend to contextual elements of a visual scene. Regarding the attention to others’ activities, children from subsistence-based farming communities often observe several activities simultaneously, while children from urban Western contexts focus on activities sequentially. Here we assessed 144 5-year-old children from three prototypical cultural contexts (urban Germany, rural Cameroon, urban Japan) to investigate variations in attention across a variety of tasks. Attention to the elements of a visual scene was assessed in an optical illusion task, in picture descriptions and an eye-tracking paradigm. Attention to and learning from others’ activities was assessed in a parallel action task and a rule-based game. Some tasks indicated higher context-sensitive attention in urban Japan, while other findings indicated higher context-sensitive attention in urban Germany. Levels of parallel attention and learning from others’ activities were lower in rural Cameroonian children compared to the urban samples. Across tasks, the visual attention measures were unrelated. These findings substantiate that culture has a profound influence on early cognitive development, already in the preschool years. Furthermore, they raise critical questions about the early origins of cultural specificities in attention and the generalizability of attention phenomena beyond specific tasks and populations.

## Introduction

Human basic cognitive processes differ strongly between cultures. A specific interest lies in the developmental origins of cultural differences in visual attention in the childhood years [1–4]. Two major lines of research from the past decades revealed cultural differences in the attention to visual scenes (for reviews, see [1, 2]) and to others’ activities when learning from others (for reviews, see [3, 4]). Thus far, both lines of research were investigated independently and focused on different age groups and different cultural contexts. In the present study, we developed a set of tasks to assess diverse aspects of children’s visual attention and employed these tasks in three prototypical cultural contexts (urban Germany, rural Cameroon, urban Japan). The main objective was to assess multiple indicators of children’s attention to visual scenes and the activities of others in order to shed new light on cultural differences in diverse aspects of visual attention, and how they may be related to each other in the preschool years.

Regarding the attention to visual scenes, there is a research tradition that focuses on educated urban middle-class populations in Western and Eastern cultural contexts and describes two prototypical attention styles [2]. An analytic style with a focus on focal objects and their properties (i.e., low context-sensitivity), described for people from Western cultural contexts, and a holistic style with a higher sensitivity for the context and the relations between elements in a scene (i.e., high context-sensitivity), described for East Asian adults. For example, Masuda and Nisbett [5] found that US-Americans tended to report a focal fish swimming in an aquarium, while Japanese participants reported more details from the background, including plants and smaller animals, when describing a picture. Similar differences were found in visual attention processes as measured by participants’ gaze behavior [6, 7]. For example, Chua and colleagues [6] presented pictures with a focal object on a background (e.g. a tiger in the woods) and recorded the gaze behavior of Chinese and US-American university students. They reported that Chinese students spent more time looking on the background than US-American students. In the same vain, East Asian participants perceive more relations between objects [8, 9] and are more easily deceived by optical illusions, when adjusting a focal element within a deceptive context [10], compared to Western participants. These attentional phenomena are assumed to index more general attention styles, with higher or lower sensitivity for contextual elements of a visual scene, which explain variation across verbal, non-verbal, and non-semantic tasks [1, 2]. Recent studies found a relation between different measures, namely picture descriptions and gaze pattern in adults [11] and a relation between picture descriptions, mnemonic measures and even visual cortical processes in early childhood [12]. Furthermore, there is evidence that differences in context-sensitive attention are socialized and culturally transmitted via verbal learning processes [11, 12].

Cross-cultural differences in holistic and analytic attention emerge roughly between 5 and 7 years of age, with evidence from picture description [13] and optical illusion tasks [11, 14, 15], as well as the Framed Line Test [16, 17]. In a study by Imada and colleagues [13], 6- to 7-year-olds (but not younger children) from Minneapolis, USA, and Kyoto, Japan, showed significant differences across several indicators from a picture description task and an Ebbinghaus illusion. A recent study compared the degree of context-dependent attention in optical illusions in children from more diverse cultural contexts, an urban sample from the United Kingdom, an urban Namibian sample, and a sample of traditional Himba children across a broad age range [14]. The researchers found that participants’ deception to the optical illusions increased in UK children in the early school years, but only between 9 and 10 years in urban Namibian children. Overall, it remained at a very low level in traditional Himba children across the investigated age range, from as early as 3 years of age into adulthood. The authors explained these effects mainly by urban environmental factors as well as different levels of experience with print media. Overall, these findings show that culture-specific attention styles start to emerge in the late preschool years, before the 6th year of age, and further increase in the years thereafter.

In another research tradition in developmental psychology, influenced by cultural anthropology, Rogoff and colleagues [3, 4] have theorized and documented differences in attention and the consequences for learning by observing the activities of others in urban Western and rural subsistence-based contexts in Central America. The authors describe a distributed attention pattern for children from Indigenous-heritage communities of the Americas, spending more time shifting their attention to different simultaneous activities and learning opportunities, compared to children from Euro-American urban middle-class families, who show a more focused, sequential attention pattern when learning from others. For instance, when learning a new activity, triads of 6- to 10-year-olds from Indigenous-heritage regions of Mexico were often keenly observing the model and the other child and distributed their attention between multiple events simultaneously. Euro-American children showed a rather focused attention style, focusing their attention on one event [18–20]. According to Rogoff and colleagues, the keen observation of others’ activities, typical reported for subsistence-based communities, also has implications for observational learning. For example, 5- to 11-year-old Indigenous-heritage children from Guatemala were better at attending to ongoing events, compared to Euro-American children. They were given a distractor toy and were told to wait, while the experimenter demonstrated how to produce a toy animal to another child, Guatemalan children were more often attending to both the distractor toy and the ongoing activity simultaneously and, as a consequence, were better at learning the demonstrated action sequence. Namely, they needed less support when given the chance to reproduce actions on a toy animal one week later [21]. Rogoff also proposes that children in these contexts, keenly attending to the activities of others, are also better at extracting conventional rules and regularities by observing others’ activities [3].

Rogoff and colleagues explain these culture-specific attention patterns when observing the activities of others by different types of learning opportunities [22]. Children from Indigenous-heritage communities typically participate in the life of adults and learn by community participation (keen observation of and pitching into the activities of others). In contrast, learning in the US is often based on direct teaching and segregated from everyday life, which becomes specifically apparent in the context of formal education.

Both lines of research describe cultural differences in the way children attend to different aspects of their environment. However, both lines of research are disparate and rely on very different theoretical assumptions and empirical findings from different cultural contexts, comparing urban Western with urban Eastern cultural contexts or urban Western with rural subsistence-based communities. The main purpose of the current study is to assess analytic and holistic visual attention [1, 2] together with sequential and distributed attention [3, 4] and potential consequences for learning from others’ activities. Specifically, we were interested in cultural differences in attention to visual scenes and the activities of others which already exist in the preschool years, before children have been influenced by formal schooling. Concerning schooling, it has been argued that it affects visual attention and learning from others in the sense that it leads to more focused and sequential patterns of attention and generally encourages direct teaching and learning at the expense of distributed attention to others’ activities [4].

We selected three cultural contexts, each representing an often-studied prototypical cultural contexts. These were urban middle-class samples from Western and East-Asian cultures, a selection informed by the first line of research, and a sample of children living in a subsistence-based farming ecology in rural Cameroon, also an often studied and well-described cultural context [23]. The characteristics of the latter nicely map onto cultural contexts described by Rogoff, especially in terms of children’s education through participation in communal endeavors and adults’ everyday activities. In developing their argument, Rogoff and colleagues repeatedly draw on findings from Indigenous-heritage, subsistence-based communities outside the Americas, mainly findings from Sub-Sahara Africa and Asia, with low levels of formal education [3, 4, 23].

These contexts were selected because they capture the essence of those included in previous research (e.g. [1, 3]). The urban German middle-class represents a prototype of an individualistic [24] (or autonomous [25]) cultural context, with small family and household sizes (mainly the nuclear family) and high levels of formal education. Parents are occupied in professional jobs, and their children usually attend the kindergarten. Children furthermore engage in organized hobby groups, and possess a large variety of advanced toys, including computer games. The focus of parental behavior and socialization goals lies on dyadic interactions and individual development, such as making choices independently [25, 26]. The lifeworld of children in Japan looks very similar in terms of the ecology (urbanization, education, wealth, modernity), however, the social structure is often referred to as interdependent. This is, personal relationships and socialization goals are characterized by stable, often life-long social bonds between family members and friends, which come with specific role obligations and entitlements [27], manifesting in an interdependent self-concept [23, 24]. Regarding the environment, Japanese urban contexts are marked by higher complexity [28] (e.g., indicated by pictures of scenes from cities in Japan and the United States). The lifeworld of children from the Nso culture in rural Cameroon differs from those of Western and Eastern urban contexts in a number of ways. Children grow up in large, extended family settings in subsistence-based villages, dominated by a relational cultural model [23]. Most parents are farmers and engage their children in household tasks and fieldwork from early on [26, 29]. From age 4, most children attend preschool in the mornings, while their parents work on the fields outside the village. Primary socialization goals are obedience and taking over responsibilities associated with social roles in hierarchical social relationships.

A large body of research on the cultural differences in human basic cognitive functions focusses on the early school years and the years thereafter. In the present study, we aimed to understand cultural differences in visual attention with no or only minimal schooling experiences (i.e., the preschool in Cameroon). Furthermore, we aimed to understand the relation between different tasks on attention to scenes and other’s behavior and the generalization of phenomena beyond previously studied cultural contexts.

Towards this end, we adapted and developed a set of tasks to assess attention to visual scenes and others’ behavior in Münster (urban Germany), Banten (a farming village in rural Cameroon) and Kyoto (urban Japan), at the age of five. To obtain multiple measures for children’s holistic and analytic attention (i.e., high or low context-sensitivity), we employed classical tasks which assess children’s context-sensitive attention, namely an optical illusion task, an eye-tracking paradigm, and a picture description task. To measure children’s attention to others’ activities, we measured their learning performance for actions that occur in parallel and by observing the conventions of a rule-based game. First, children saw two action sequences simultaneously (manual production of a toy), their gaze was recorded, and they could reproduce both action sequences afterwards. Second, children saw two actors playing a rule-based game that included sorting rules (objects to locations) and hand activities, before they played the game with the experimenter to assess their learning performance.

Drawing on the two lines of research outlined above, we hypothesized that visual attention measures tend towards the holistic style in Kyoto as compared to Münster, and, based on the findings from the optical illusion tasks [14, 15], a more object-focused attention style for Banten, compared to children from the urban contexts. Furthermore, we expected higher levels of distributed attention when observing parallel activities in the village of Banten as compared to the Western urban context in Münster and, possibly, also compared to Japan. Regarding the two different theoretical views on children’s attention across cultures, namely analytic versus holistic and sequential versus distributed attention, we aimed to explore if these measures would be associated with each other across different tasks and domains (i.e., attention to visual scenes and the activities of others), in the preschool years.

## Results

We assessed 144 5-year-old children (*M* = 5;5 years, *SD* = 0;4 in Germany, *M* = 5;6 years, *SD* = 0;4 in Cameroon, and *M* = 5;8 years, *SD* = 0;3 in Japan) from Münster (urban Germany; *n* = 43), farming villages in Banten near Kumbo (rural Cameroon; *n* = 52), and Kyoto (urban Japan; *n* = 49). The age of the children was significantly different between cultures, *F*(2, 141) = 8.62, *p* < .001, namely children from Japan were older then children from Germany and Cameroon, both |*t*| > 2.53, *p* < .013. However, overall, age did not correlate with the 14 dependent measures reported throughout the analyses in any of the three cultures (i.e., only 1 out of 42 correlations reached *p* < .05). Thus, age was not entered as a covariate in the subsequent analyses. Proportions of girls and boys (60% girls in Germany, 45% girls in Cameroon, and 52% girls in Japan) did not differ between the three samples, χ^2^ = 2.22, *p* >.25.

Note that not all children completed the full set of tasks and that, for each task, analyses are based on the complete subsets from each culture (find details about the procedure, analyses, and exclusion criteria in the Materials and Methods section below.) All data reported in the manuscript are available in the Supporting Information (S1 File).

### Cultural contexts

Parameters of children’s family contexts are summarized in Table 1. There were significant differences in the number of siblings, with a higher number of siblings in rural Cameroon compared to two urban contexts, both |*t*| > 11.62, *p* < .001, also reflected in the people living in the household, both |*t*| > 13.67, *p* < .001. In Germany, the majority of mothers and fathers worked in the professional jobs (62.8% and 74.4%, respectively), in Cameroon most parents were farmers (mothers: 92.5%, fathers: 89.6%), and in Japan mothers were mostly house wives (41.8%) with husbands being office workers (51.0%).

**Table 1:** Family contexts.

Variables	Germany	Cameroon	Japan	*F*	*p*
Age mother (years)	39.9 (5.3)	37.7 (7.7)	40.0 (3.8)	1.11	>.25
Number of siblings	1.1 (0.8)	5.1 (2.1)	1.2 (0.9)	8.62	< .001
People in household	4.0 (1.0)	6.6 (2.0)	4.1 (0.8)	168.32	< .001
Mother, years of school	16.0 (3.1)	7.1 (2.8)	15.5 (1.8)	174.92	< .001

Note. The table presents means with standard deviations in parentheses.

We tested for potential correlations between three socio-demographic variables, which we assessed from all children (number of siblings, people in the household, years of maternal education, see Table 1). The correlation of these three measures with all 14 dependent measures from the three different tasks (in 3 cultures) revealed that only 8 out of 126 correlations (i.e., 6.35% of all correlations) were significant at the level of *p* < .05, which corresponds pretty much to the chance level. Thus, these variables were not included as covariates in the subsequent analyses. This is, since the potential covariates (age of the mother, number of siblings, people in the household, years of maternal education) were not independent of the group variable culture (independent variable), and there was no major influence of these potential covariates on the dependent variables, the preconditions for an analysis of covariance (ANCOVA) were not met ([30], p. 200).

### Optical illusion task

**Fig 1:**
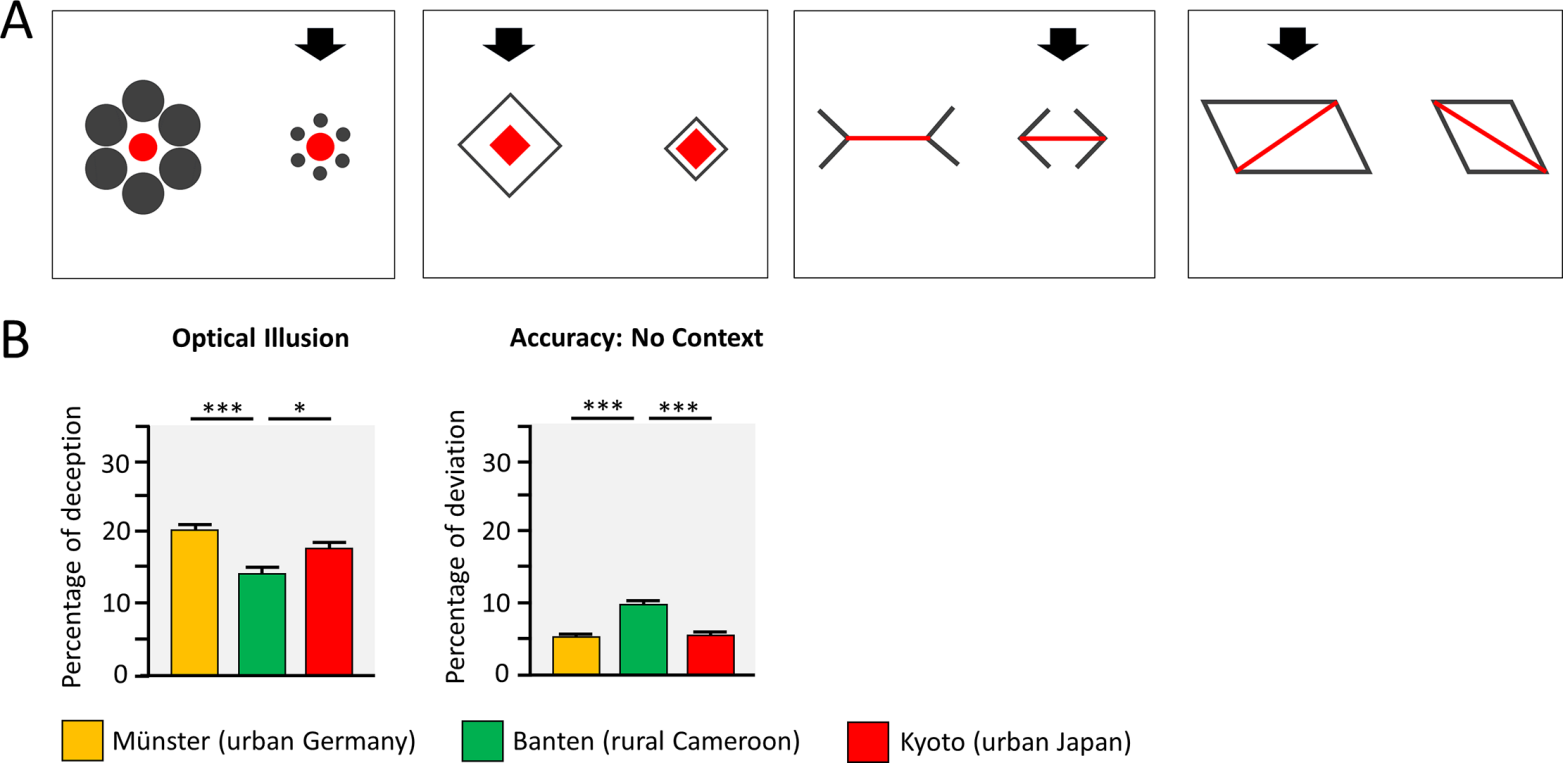
Optical illusion task. (A) The four optical illusions. The red element had to be adjusted at the side indicated by the black arrow. (B) Group comparisons indicate the results of t-tests, following significant main effects. * *p* < .05, ** *p* < .01, *** *p* < .001, Bonferroni corrected.

We tested children’s deception by context information with an optical illusion task [11–15] for four illusions (two Ebbinghaus illusions, Müller-Lyer illusion, Sander illusion), see Fig 1A, each illusion presented twice. Before the illusion task, the same red elements were shown without the gray, illusory contextual elements, to obtain a measure of children’s accuracy.

In a first step, the illusion scores were entered into a repeated-measures ANOVA with task (4 tasks) as within factor and culture (3 contexts) as between factor. We found a main effect of task, *F*(3, 414) = 131.59, *p* < .001, *η^2^* = .49, and culture, *F*(2, 138) = 11.76, *p* < .001, *η^2^* = .15, but no culture x task interaction, *F*(6, 414) = 1.21, *p* = .301, *η^2^* = .02. Urban German and urban Japanese children were deceived to a higher degree (*M* = 20.1% and *M* = 17.7%) than children from rural Cameroon (*M* = 13.9%), see Fig 1B. Mean deception values, separated by task were: Ebbinghaus, circle: 9.3%; Ebbinghaus, diamond: 8.0%; Müller-Lyer: 24.1%; Sander: 27.1%. We further assessed the accuracy of the adjustments of the red shapes in control trials, without context. Urban German and urban Japanese children were more accurate (*M* = 5.2% and *M* = 5.4%) than children from rural Cameroon (*M* = 9.5%), *F*(2, 138) = 59.41, *p* < .001, *η^2^* = .46, see Fig 1B.

In a second step, we explored the correlations between the scores of the different illusions, to see if they would assess the same perceptual phenomenon. This revealed that the scores from both versions of the Ebbinghaus illusion were correlated, consistently in all three cultures (urban Germany: *r* = .44, *p* = .004; rural Cameroon: *r* = .23, *p* = .115; urban Japan: *r* = .34, *p* = .015). Likewise, the scores from the Müller-Lyer and the Sander illusion were correlated (urban Germany: *r* = .29, *p* = .063; rural Cameroon: *r* = .35, *p* = .014; urban Japan: *r* = .30, *p* = .032). All other correlations between the optical illusions were low (urban Germany: both |*r*| < .16, *p* > .314; rural Cameroon: |*r*| < .11, *p* > .461; urban Japan: |*r*| < .11, *p* > .453). Thus, these two pairs of illusions seem to assess independent perceptual processes, which may be described as field dependence (Ebbinghaus illusions) and sensitivity to carpentered corners (Müller-Lyer and the Sander illusion).

### Picture description task

**Fig 2:**
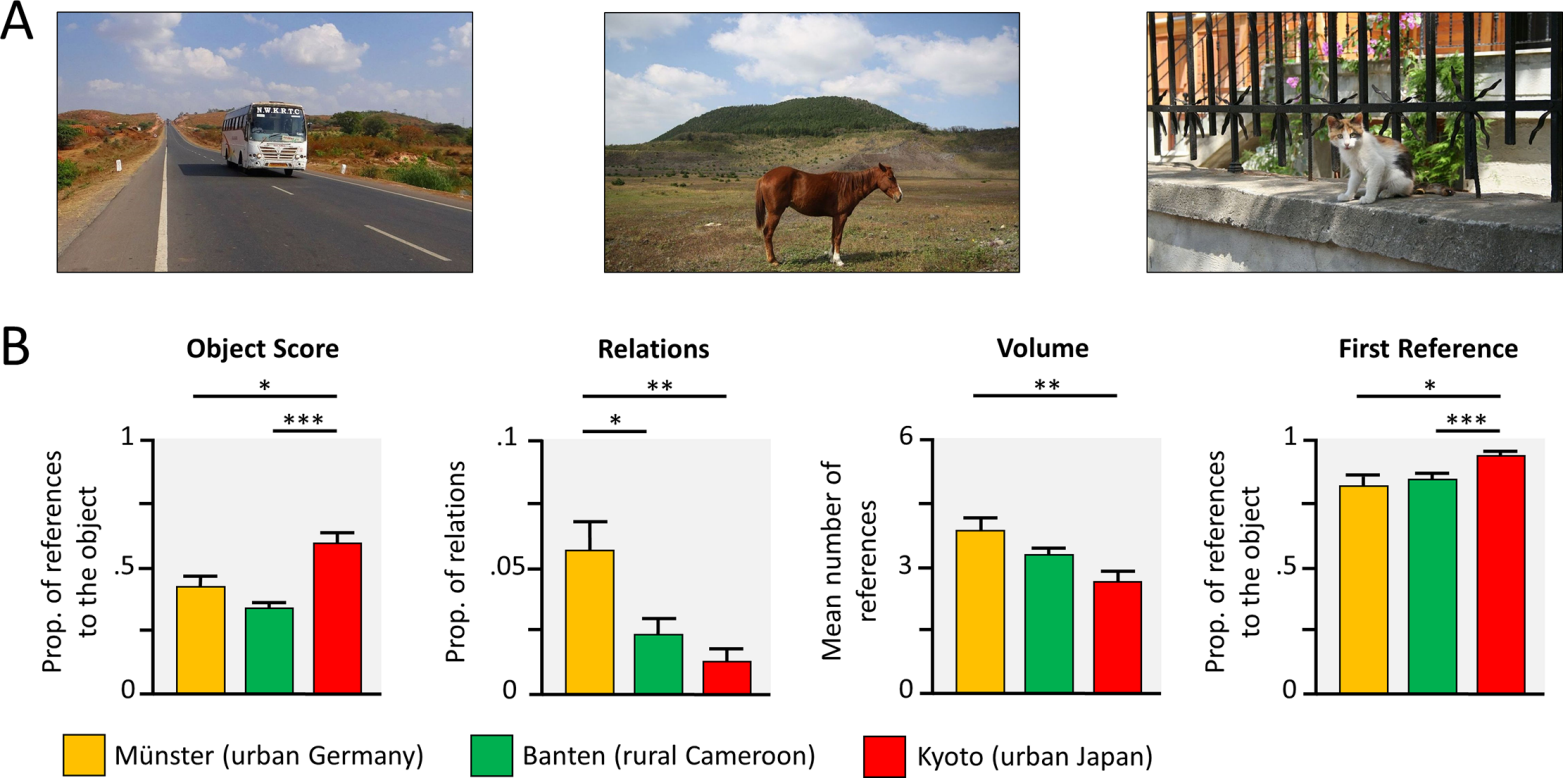
Picture description task. (A) Children described pictures with a focal object to the experimenter. (B) Group comparisons indicate the results of t-tests, following significant ANOVAs. * *p* < .05, ** *p* < .01, *** *p* < .001, Bonferroni corrected.

Another task to assess context-sensitivity in terms of explicit attention to focal and background elements and their features are picture description tasks [11–13]. Here, children described pictures with a focal object (animals and means of transport), in front of a simple background (e.g., natural scenes, roads and buildings), see Fig 2A, to the experimenter.

The relative number of references to the object, compared to the references to the background (object score) differed between cultures, *F*(2, 121) = 14.95, *p* < .001, *η^2^* = .20, and was higher in urban Japanese children, compared to both other samples, see Fig 2B. German children uttered a higher proportion of relations between the elements of a scene, compared to rural Cameroonian children (*M* = .057 versus *M* = .024 relations per picture) and Japanese children, where relations were almost absent (*M* = .013 relations per picture), *F*(2, 121) = 7.72, *p* < .001, *η^2^* = .11.

Part of these findings may be explained by differences in how talkative children were, *F*(2, 121) = 7.63, *p* < .001, *η^2^* = .11. Picture descriptions were most verbose in urban Germany (*M* = 3.9 references to object or background and their features), followed by rural Cameroon (3.3 references) and then urban Japan (*M* = 2.7 references), and differed significantly between Germany and Japan. Correlations between number of references (i.e., volume) and the dependent variables (object score and relations) revealed a highly negative correlation between object score and volume across samples, all *r* < -.75, all *p* < .001, but no correlation with the number of relations, all *r* < .15, all *p* > .353. Therefore, we also looked at the object score for the first reference made by the participants, i.e., the percentage of picture descriptions that started with the object and not the background. Similar to the results of the object score, this analysis revealed a significantly higher proportion of first references to the object in urban Japanese (*M* = .95) compared to urban German (*M* = .82) and rural Cameroonian children (*M* = .85), *F*(2, 121) = 6.53, *p* = .002, *η^2^* = .10.

### Eye-tracking task

**Fig 3:**
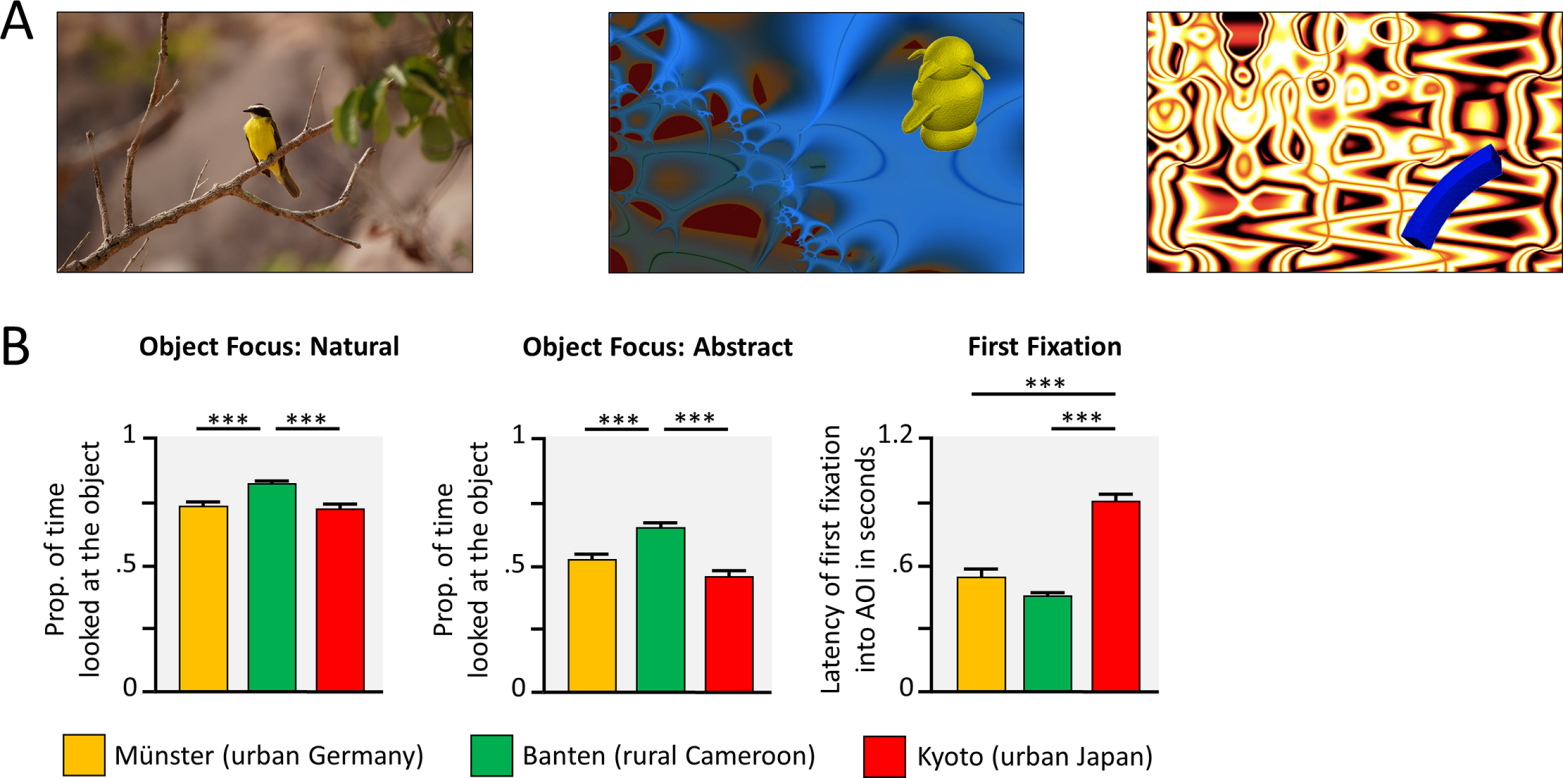
Visual attention in an eye-tracking task. (A) Children saw natural pictures with a clear object and background, as well as abstract objects background combinations. (B) Group comparisons indicate the results of t-tests, following significant ANOVAs. * *p* < .05, ** *p* < .01, *** *p* < .001, Bonferroni corrected.

To assess early visual processing of scenes, similar pictures to those in the picture description task where presented, participants’ gaze behavior was recorded [6, 11]. Children saw real pictures which displayed objects in front of a simple background and abstract (non-semantic) pictures with artificial objects [31] on abstract backgrounds [32], see Fig 3A. These where included because they were unfamiliar to children from all contexts.

In a first step, we entered the proportion of (1) the total looking time spent exploring the object and (2) the latency of the first fixation to the object into two independent ANOVAs with the within factor picture type (natural, abstract) and culture as a between factor. Both ANOVAs revealed a main effect of picture type, latency: *F*(1, 127) = 207.13, *p* < .001, *η^2^* = .62, looking time: *F*(1, 127) = 414.49, *p* < .001, *η^2^* = .77, and culture, latency: *F*(2, 127) = 72.60, *p* < .001, *η^2^* = .53, looking time: *F*(2, 127) = 46.92, *p* < .001, *η^2^* = .10, and the looking time on the object (but not the latency score) revealed a culture x picture type interaction, *F*(2, 127) = 7.70, *p* < .001, *η^2^* = .11. Given this interaction, we further explored the cultural differences in looking time in subsidiary ANOVAs, split for the picture types. For natural pictures, rural Cameroonian children looked at the objects in each picture longest (object focus: *M* = 82% of the looking time), compared to urban German children (*M* = 73%) and urban Japanese children (*M* = 72%), see Fig 3B, *F*(2, 127) = 70.17, *p* < .001, *η^2^* = .53, and, *F*(2, 127) = 24.46, *p* < .001, *η^2^* = .28, respectively. For the abstract pictures, there were significant differences in the object focus between cultures, *F*(2, 127) = 34.85, *p* < .001, *η^2^* = .35, again, with a higher focus on the object in rural Cameroon, (*M* = 65%), compared to urban Germany (*M* = 52%), and urban Japan (*M* = 46%). The first fixation onto the object, collapsed over stimulus type, was significantly later in Japanese (*M* = 907 ms) compared to German (*M* = 548 ms) and rural Cameroonian children (*M* = 459 ms), *F*(2, 127) = 72.60, *p* < .001.

Furthermore, we explored the correlations between the scores of the different measures, to see if they would assess the same perceptual phenomenon. Correlational analyses revealed that all four measures were closely related in urban Germany (all six correlations: |*r*| > .31, *p* < .059, at least at the level of a trend). However, the correlations were less consistent in rural Cameroon and urban Japan, where correlations were only found between the two measures (Object focus and first fixation) within the two stimulus categories, namely for normal pictures in Japan (*r* = -.31, *p* < .042), and for abstract pictures in Cameroon (*r* = -.51, *p* < .001) and Japan (*r* = -.49, *p* < .001).

### Parallel actions task

**Fig 4:**
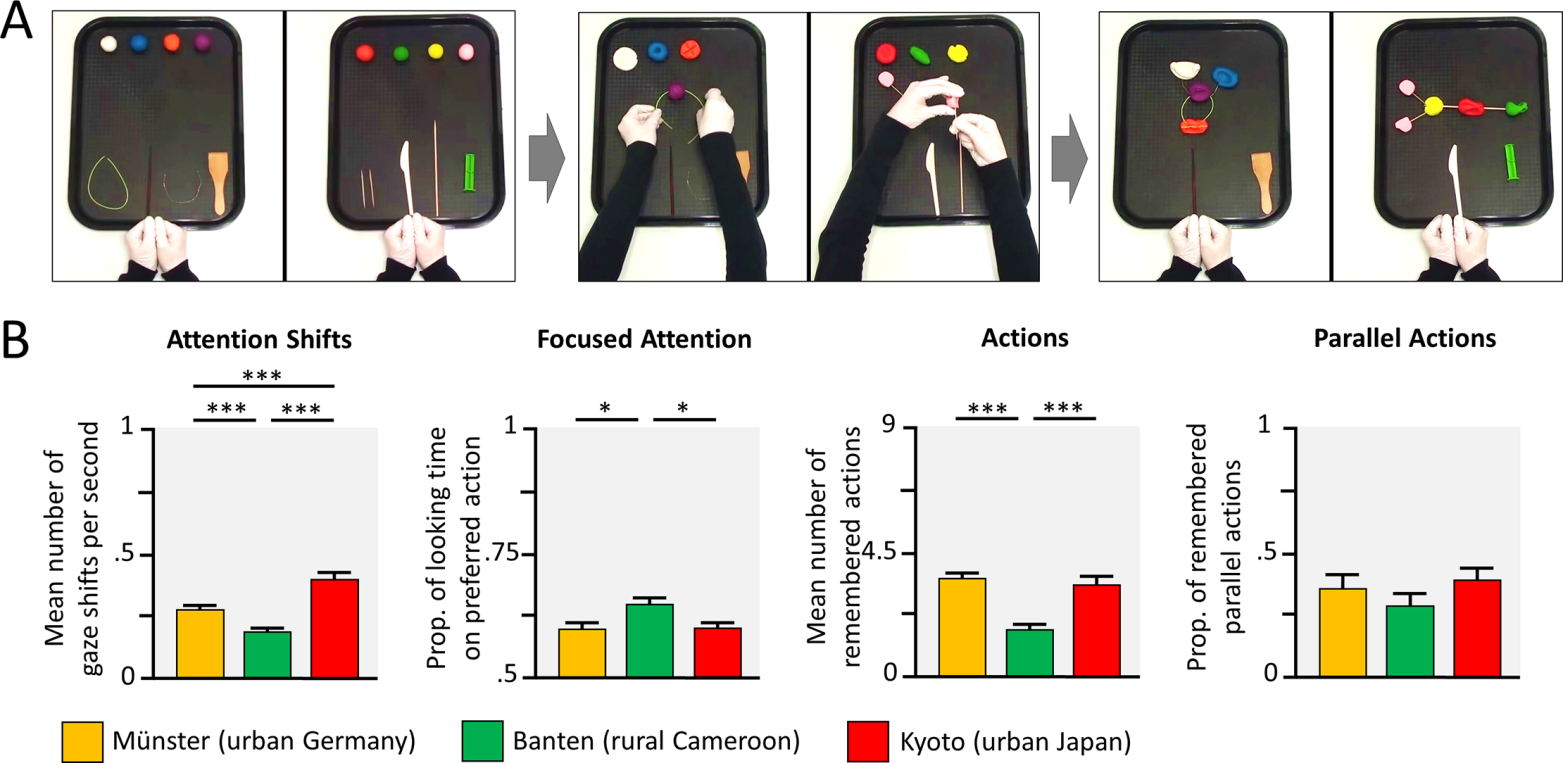
Attention to and reproduction of parallel action sequences. (A) Children saw a video, in which 2 actions sequences were performed in parallel, and their gaze was recorded. Later on the reproduction of actions was analyzed. (B) Group comparisons indicate the results of t-tests, following significant ANOVAs. * *p* < .05, ** *p* < .01, *** *p* < .001, Bonferroni corrected.

We aimed to capture children’s distributed attention, that is, the extent to which children are able to attend to two activities in parallel, more rigorously than previous studies [19–21]. Therefore, we developed a video-based task, with two action sequences shown simultaneously, see Fig 4A and S2 File. As a direct measure of distributed attention, we recorded the gaze behavior of the children and, as an indirect measure of distributed attention, children were given the opportunity to reproduce the two handicrafts afterwards and their performance was coded.

The number of attention shifts from one action sequence to the other was higher in urban Japanese, compared to urban German, and compared to rural Cameroonian children, and attention shifts were also significantly more frequent in urban Germany compared to rural Cameroon, see Fig 4B, *F*(2, 126) = 35.54, *p* < .001, *η^2^* = .36. Furthermore, rural Cameroonian children focused more on one of the two action sequences (*M* = 64.9%), compared to the urban German (*M* = 60.0%) and to the Japanese children (*M* = 59.9%), *F*(2, 126) = 5.71, *p* = .004, *η^2^* = .08.

With regard to the number of actions reproduced, children from rural Cameroon exhibited fewer of the nine action sequences (*M* = 1.75) than children from urban Germany (*M* = 3.65) and Japan (*M* = 3.44), *F*(2, 132) = 20.73, *p* < .001, *η^2^* = .24. However, when looking at the proportion of both parallel actions out of all reproduced actions, in the number of all actions, there were no differences between cultural contexts, *F*(2, 129) = 1.22, *p* = .300, *η^2^* = .02.

Correlational analyses, to see if the different measures would assess similar attention processes, revealed close associations between the rate of attention shifts and the distribution of attention (Germany: *r* = -.42, *p* = .009; Cameroon: *r* = -.44, *p* = .002; Japan: *r* = -.21, *p* = .164), this is, the more they shifted their gaze between action sequences, the more equal they distributed their gaze time between both actions. Noteworthy, this close association may be due to the close dependency of both measures, with more attention shifts allowing an more equal distribution of attention towards the two action. Furthermore, the number of reproduced actions was closely related to the proportion of parallel actions children reproduced (Germany: *r* = .74, *p* < .001; Cameroon: *r* = .24, *p* = .108; Japan: *r* = .62, *p* < .001). However, there were no correlations between the visual attention measures and the learning measures (all |*r*| > .26, *p* > .13). Again, this may be because both measures are interdependent, with more actions reproduced leading to higher probability of parallel actions to be reproduced.

### Rule-based game

**Fig 5:**
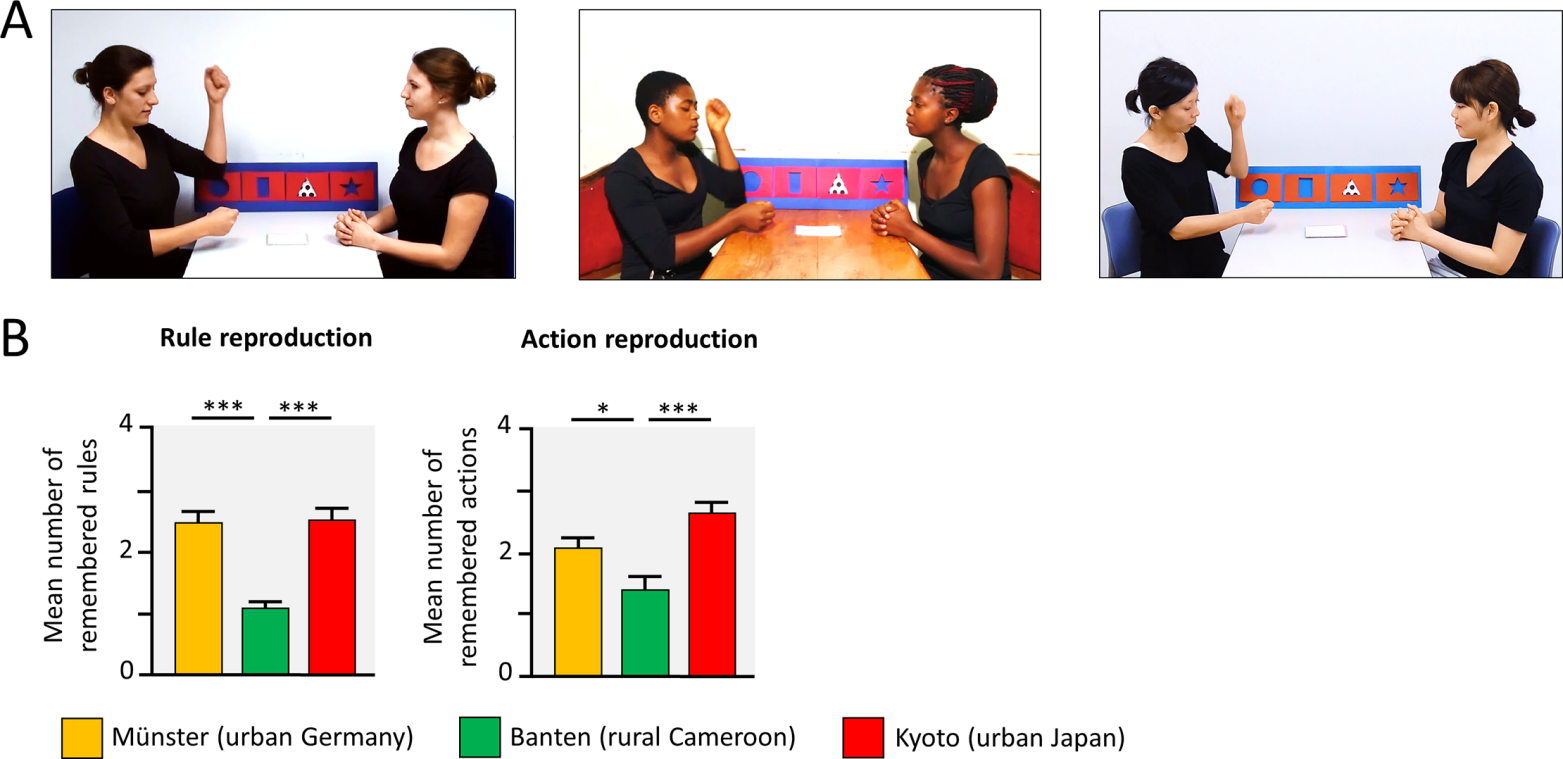
Learning conventions in a rule-based game. (A) Children saw a video, in which 2 players played a rule-based game including 4 rules and 4 actions. Later on, their reproduction for the rules and actions was tested, when playing the game with the experimenter. (B) Group comparisons indicate the results of t-tests, following significant ANOVAs. * *p* < .05, ** *p* < .01, *** *p* < .001, Bonferroni corrected.

The rule-based game was designed to assess the competence to infer conventional rules based on attending others playing a game [1, 2]. Specifically, we wanted to test children’s ability to understand the rules underlying the observed actions of a social game. Children saw a video with two people playing a rule-based game, see Fig 5A, before child and experimenter played the game together.

Rural Cameroonian children acquired fewer rules (*M* = 1.06 remembered rules), compared to German (*M* = 2.44) and Japanese children (2.48), *F*(2, 139) = 24.81, *p* < .001, *η^2^* = .26, see Fig 5B. Action reproduction differed significantly between all three cultural contexts with the highest number of reproduced action by urban Japanese children (*M* = 2.64), and higher action reproduction skills in urban Germany (*M* = 2.07), compared to rural Cameroon (1.39), *F*(2, 139) = 11.74, *p* < .001, *η^2^* = .15. Both measures were correlated in the rural Cameroonian sample (*r* = .34, *p* = .017) but not in the two urban samples (both |*r*| < .03, *p* > .87).

### Correlations between the measures between the different tasks

Regarding the between-task correlations within each cultural context, there was no consistent correlational pattern. This was indicated by the fact that, first, no single correlation between two measures reached significance across all three contexts and, second, that less than 10% of all correlations (uncorrected) were above |*r*| = .30.

## Discussion

The aim of the present study was to investigate similarities and differences in the visual attention to scenes [1, 2] and to the activities of others [3, 4], in preschool children from different cultural contexts. Towards this end, we investigated these phenomena across several tasks in three prototypical cultures (urban Western, urban Eastern and a rural subsistence-based village contexts). First, this was to assemble tasks which assess different aspects of children’s visual attention processes, namely analytic and holistic attention [1, 2], as well as sequential and distributed attention [3, 4]. Second, we assessed these tasks in three cultural contexts that combined the cultural contrasts of previous studies, namely Western vs. East-Asian urban middle-class and Western urban middle-class vs. Non-Western subsistence-based farming ecologies. This also allowed us to test the consistency of different aspects of children’s attention pattern across tasks and cultures.

Taken together, the findings of the present study indicate that basic cognitive functions vary highly between cultures, already in the preschool years. At the same time, as outlined in more detail below, our findings were much more heterogeneous than former literature suggested and, despite the significant cross-cultural differences in attention to scenes and others’ activities, the different measures were not related to each other.

### Cultural differences in attention to visual scenes

Our main assumption was that Japanese (urban East-Asian) children would show greater levels of holistic attention, compared to German (urban Western) children. The results were mixed, some pointing into the expected direction and other pointing into the opposite direction Taken together, like in former studies, the results from the optical illusion task did not differ between cultures and the results from the eye-tracking task show first hints for the classical differences between urban Western and urban Eastern contexts. Furthermore, results were contrary to the classical cultural pattern in the picture description task, pointing towards higher levels of context-sensitive attention in Germany compared to Japan. Furthermore, there were no consistent correlations between tasks in the preschool age, which is also consistent with former studies, reporting correlations only for older children and adults [11, 12].

The visual attention of children from rural Cameroon differed from both urban contexts and was characterized by a high object focus across tasks. In this sample, we found the lowest level of deception in the optical illusion task. Furthermore, Cameroonian children made the first saccade on the object most rapidly and spent more time exploring the object than both other samples. In the picture description task, Cameroonian children fell somewhere between the other two samples. The reduced deceivability of rural Cameroonian children is generally in line with ecological accounts of optical illusions, which emphasize the importance of the experiences with carpentered corners for the effect of optical illusions like the Müller-Lyer and the Sander illusion (cf. [33]). Specifically, the present findings complement recent studies conducted in another rural village context, namely in traditional Himba people [14], where context-sensitivity in the Ebbinghaus task was very low across the whole life span. Thus, cultural differences in context-sensitive attention are present already in the preschool years, when they are not yet found in the classical eastern western comparison (see [13, 16] and above). The higher object focus in the eye-tracking paradigm is consistent with the low level of context sensitivity in the illusion tasks. Alternatively, this may also reflect an unfamiliarity-effect, because the objects shown here, both real and abstract, are less common in the Nso children’s lifeworld and may have led to an increased interest.

One potential explanation for the low levels of context-sensitivity found in the picture description task would be that this sample of Japanese urban dwelling children were non-traditional or differed in some other way from the typical Japanese samples in other studies [1, 2]. However, the participants came from the same population as in the study by Imada and colleagues [13], where context-sensitive attention, pooled across several tasks, including picture descriptions, emerged in the school years.

Overall, these findings suggest that visual attention towards focal or contextual elements of a scene is influenced by the cultural context. However, in the early childhood years it seems not yet to be a consistent construct that aligns visual attention across different types of tasks (e.g., verbal, non-verbal, semantic, abstract). Potentially, the culture-specific context-sensitivity pattern reported in literature may override the differences identified here later in development [11, 12, 13, 34], possibly via verbally guided learning processes [11, 12, 35], for example, as found in formal schooling. The differences in visual attention identified here, for the preschool years, may be based on different cultural and environmental learning experiences. For example, they may rather reflect children’s basic familiarity with the stimulus materials and stimuli, rather than culturally transmitted attention styles. In line with this proposal, see recent studies by Köster and colleagues [11, 12], for similar inconsistencies in context sensitive attention in 5-year-olds, which develop in more coherent pattern from around 7 years of age.

### Cultural differences in in attention to others’ activities

Regarding children’s attention to others’ activities, we found a different pattern than hypothesized. Specifically, in the parallel action task, children from rural Cameroon focused their attention rather on one of two activities and shifted their gaze between scenes at a lower rate than children from both urban contexts. Furthermore, their learning performance was at an overall lower rate. Similarly, in the rule-based game, rural Cameroonian children performed fewer rules and actions compared to urban German and Japanese children. The comparison between urban Germany and urban Japan did not reveal many differences, but there were more frequent shifts of attention in Japan then in Germany, when observing two action sequences in parallel.

Interestingly, the attention pattern towards parallel actions, namely a more unilateral attention on one of the two activities in rural Cameroonian children, resembles the more object-focused attention of Cameroonian children found in the visual attention task. Although the attention and learning pattern was different from previous studies [3, 4], it may be speculated that the ability to distribute attention to various aspects of a visual scenes or when observing others’ activities allows children to grasp more details about the activities and conventions of others. Here, this may explain higher scores of distributed attention as well as learning scores in Germany and Japan. However, within cultures, we found no consistent correlations between the tasks assessing the attention to visual scenes and the activities of others, despite moderately large sized samples.

### Differences in visual attention from a lifeworld perspective

Most importantly, the task testing visual attention to scenes employed here were, thus far, mostly applied in Western and Eastern urban contexts, and the materials used to test visual attention to others activities used materials certainly more familiar to German and Japanese children. Furthermore, the form of presentation, namely on a computer screen, was not known to Cameroonian children. Thus the present results, and in particular the learning performances for others’ activities have to been interpreted in the light of the very different lifeworld’s and learning contexts, which children from the different sample reported here grow up in.

We outlined the contrast of the lifeworlds of the prototypical contexts chosen here in the introduction. In the tenet outlined above, the manifold variations in children’s visual attention between cultures identified here, are very likely due to the different experiences children make in their environment for their first five years of life. In this sense, we conceptualize early cultural differences in visual attention as a results of versatile learning experiences. At least, common dichotomous perspectives, based on one or a few experimental tasks and the comparison of a two cultural contexts, do not account for the variations between and within the cultural contexts identified here.

In this sense, the present results also illustrate the great challenges for the development of experimental materials, which may capture different aspects of children’s attention in a culturally fair way. For example, regarding rural Cameroon, we chose very simple materials for the construction of the toys and very basic material and actions in the social game. However, it may still be less common for children from Cameroon to tinker handicrafts or to learn rule-based games or, critically, to acquire novel information from a screen, instead of a live interaction. Noteworthy, although the children watched the two tasks simultaneously, they were given the construction tasks sequentially, which may be more typical in the urban contexts. Regarding the pictorial stimuli, Cameroonian children did only recognize and label about half of the objects assessed in the picture description task (and only those were taken into account in the analyses). Similarly, abstract stimuli might be more familiar to children in Münster and Kyoto. Thus, the present findings on learning from others may demonstrate that cognitive functions are inextricably knit to everyday visual experiences and materials (cf. [36]).

### Future directions

By assessing visual attention with multiple tasks and methods and in novel cultures, the present findings add an important piece of puzzle to the existing literature. Because all measures indicated high cross-cultural variation at an early age already, there does not seem to be much generalizability beyond the specific contexts and tasks. Thus, more generally, the field would benefit from a higher variation of cultural contexts and experimental tasks assessed in these contexts. Beyond the description of variations, it would also be highly valuable to more thoroughly assess environmental variables and cultural transmission processes (e.g., socialization), which may better elucidate the developmental origins of these variations.

We selected the preschool age in the present study to avoid the influence of formal schooling. This age may yet have been too early for attention processes to develop into a coherent culture-specific pattern of attention, as indicated by the correlation of different measures of context-sensitivity found in older children [12] and adults [11]. Specifically, recent findings highlight the role of language in cultural transmission processes [11, 12, 35]. To better understand the enculturation process of general attention styles, which may emerge later in life, future studies should be complemented by longitudinal or cross-sectional assessments, across a broader age-range.

With regard to the very different lifeworlds of children from the present study, the tasks employed here may rather reflect the activities of urban Western and East-Asian cultures. This had very likely negative consequences for the learning performance of the rural Cameroonian children. Future research may benefit from adapting some of the experimental tasks more closely to the daily activities and the materials in this cultural context. This includes children’s activities and play habits, the materials and pictorial stimuli used, as well as the form of presentation. For example, using life interactions to demonstrate activities.

It should also be noted that, besides those features of the tasks that assess the attention to the elements of a visual scene and others’ activities, each task requires additional and unique cognitive capacities and skills that may have contributed to cultural differences and compromised associations between tasks. For instance, performance in the optical illusion tasks may furthermore depend on inhibitory control, and both the picture description and eye-tracking tasks may depend on the familiarity with the natural, but also the abstract stimuli used. Finally, the reproduction tasks for the observed activates require advanced memory and, in the case of the artcraft, manual skills. Although we tried to keep these requirements as low as possible, we cannot preclude that these have caused some of the inconsistencies found in the present study. Thus, for future research it would be desirable to assess the diverse skills and possibly experiences which contribute to the differential response pattern in different tasks in early childhood.

Taken together, the present study highlights the general importance (i) to assess common phenomena from cross-cultural Psychology with more diverse populations and tasks, (ii) to analyze changes in a broader age range, in order to understand developmental trajectories, and (iii) to assess more thoroughly and formally specific characteristics of the eco-social environment and adapt tasks more closely to different lifeworlds. Thus, the present study underlines the necessity of further research, to elucidate theoretical accounts on ecological and social factors that contribute to inter-individual variations in cognitive development.

### Conclusion

The cultural differences in attention to the elements of a visual scene and the activities of others in the preschool years, demonstrate early differences in development between cultures, which are more diverse than common theoretical accounts suggest. Human cognitive functions and processes align with and are tightly knit to the very specific experiences, such as the learning materials and activities. At least for the phenomenon of context-sensitivity, it may only be later in life that more general attention styles shape visual processes. It is a major challenge for future research to identify the learning mechanisms and socio-ecological factors that give rise to the cultural versatility and culture-specific developmental trajectories of human basic cognitive functions in early childhood and later in life.

## Materials and methods

### Participants

We assessed 144 5-year-old children from three different cultural contexts, selected for their ecosocial profiles. Forty-three children lived in middle-class families from Münster (urban Germany), 52 children came from small subsistence-based farming villages in Banten near Kumbo (rural Cameroon), and 49 children were from middle-class families in Kyoto (urban Japan). In Cameroonian villages, children were recruited in cooperation with local schools. In Kyoto and Münster, mothers were contacted via databases from the university. Four additional data assessments from rural Cameroon were not included in the analysis, because children came from a different ethnic group and did not speak Lamnso, the local language. Informed written consent was obtained from each parent, in each context, and children gave informed assent. Furthermore, not all children completed the full set of tasks. The number of missing children is indicated for each task below.

### Ethics statement

This research was conducted in accordance with the Declaration of Helsinki and the Ethical Principles of the German Psychological Society (DGPs), the Association of German Professional Psychologists (BDP), and the American Psychological Association (APA). It involved no invasive or otherwise ethically problematic techniques and no deception (and therefore, according to National jurisdiction, did not require a separate vote by a local Institutional Review Board; see the regulations on freedom of research in the German Constitution (§ 5 (3)), and the German University Law (§ 22)).

### Stimuli and procedure

Children visited the laboratory for one experimental session. In Cameroon the laboratory was set up close to the school, whereas in Japan and Germany children visited the Laboratory of the University with a parent. Because the research focus was also on associations between tasks, but not on mean differences between conditions, assessments follow a fixed order, starting a warm-up phase, followed by the optical illusion task, the parallel learning task, the social game task, and finally the eye-tracking and the picture description task. The instructions for each task were read out to the children and children repeated the instructions prior to the start of each task. The stimuli presentation procedures were implemented in psychophysics toolbox (Version 3.0.12, on MATLAB, Version R2013a) and with the respective presentation programs in the eye-tracking tasks (Cameroon and Germany: ExperimentCenter, Version 3.5.169; Japan: Tobii Studio, Version 3.3.2). The presentation was on a notebook display and keyboard in Cameroon and a desktop Monitor and external keyboard in Japan and Germany. However, we used the same size for stimulus across cultural-contexts, 15 inch, at distance of 50–70 cm and we did not note any difficulties in the use of the keyboards in either of the two setups.

#### Optical illusion task

Four different optical illusions (two versions of the Ebbinghaus illusion, Müller-Lyer illusion, Sander illusion; see Fig 1A) were shown twice, resulting in eight trials, presented in a randomized order. An adjustable element and a reference element were colored in red and deceiving context elements were colored in gray, see Fig 1C. The red element that could be adjusted was indicated by a black arrow and could be adjusted between -35 and +35 percent, compared to the size of the reference element via two keys of a keyboard.

Children were asked to pay attention to the red elements to the same size as the reference element and to “not pay attention to the context, but only to the red element” (to avoid an relative interpretation of the task instruction). Prior to the actual task, children were instructed carefully and could practice the adjustment of a red element in one training trial. Trials were presented in a randomized order and the side of the context elements was counterbalanced.

Furthermore, as a control condition, prior to the optical illusion task, children adjusted the size of each pair of red elements for all eight trials without the presence of the gray context element. This was to assess participants’ overall accuracy and correct the degree to which children were deceived by the context [11–13].

Children’s illusion score for each task was computed by subtracting the percent of deception in the optical illusion tasks from the percent of deception in the trials without contextual information (in both conditions, with and without contextual information, we calculated the deception as the difference between the adjusted element and the red reference element). The mean deception over both trials of each illusion in percent (i.e., adjusting the left or the right shape) was used as the context-sensitivity measure for this task. For the overall accuracy, that is the precision of adjustment in trials without context, we averaged the absolute differences between the adjustable and the context element across all trials in the no context condition.

Three children were excluded from the analyses of the optical illusion task (Cameroon: *n* = 2; Japan: *n* = 1). This was due to very high deviation between the reference and the adjustable element, indicated by an accuracy value that deviated more than 2 *SD* from the sample specific mean, indicating difficulties in task comprehension.

#### Picture description task

Twenty real pictures which displayed a focal object (animals and means of transport), in front of a simple background (e.g., natural scenes, roads and buildings), see Fig 2. Pictures were taken by the first author or downloaded from a public domain database (pixabay.com). Pictures were presented for 15 s each, in a randomized order and separated by a blank screen. Children were instructed to tell the experimenter, what they see on the pictures (exact wording: “… tell me everything you see on the pictures”). While children described the pictures, the experimenter listened attentively. The final analyses are based on those 11 out of the initial 20 pictures that the Cameroonian children reliably identify and label.

Audio recordings of 15 seconds were taken for each picture and coded in MaxQDA (Version 12). Each occurrence of the following categories was coded: (a) References to the focal object and its features (e.g., camel, car is large, looks happy), (b) references to the background (e.g., desert, road) and its features (e.g., is rocky, has green leaves), and (c) any relations that were made between elements within the picture (e.g., is driving on, is looking at).

In order to quantify participants’ descriptions of the object compared to the background, we computed an object score: For each trial, all references to the object were divided by the number of all references to the object and the background. Thus, a score of 1 would indicate that a participant only talks about the object, and a score of 0 would indicate that a participant only refers to the background. These scores were averaged over all trials for each participant. Furthermore, we calculated the proportion of relations compared to all (number of relations divided by the sum of all references to the object, the background and all relations) as a second indicator for their context-sensitivity. Finally, we looked at the average volume of references (to object or background) and the proportion of first references, which referred to the object.

In Germany and Japan the coding was done by native speakers. In Cameroon the data were translated and transcribed from the local language (Lamnso) to English for later coding. To compute inter-rater reliability in the Japanese sample, 20% of the picture descriptions were translated and transcribed to German by a native Japanese, fluent in German. Inter-rater agreements were assessed for more than 20% of the data (Cohen’s kappas for were: Germany: κ _object and features_ = .82, κ _background and features_ = .79, κ _relations_ = .91; Cameroon: κ _object and features_ = .80, κ _background and features_ = .89, κ _relations_ = .98; Japan: κ _object and features_ = .63, κ _background and features_ = .81, κ _relations_ = .57, the low kappa for relations in the Japanese sample was due to the low number of relations uttered by the children).

Twenty children were excluded from the analyses of this task, due to technical errors occurring during this task (Germany: *n* = 4; Japan: *n* = 4), insufficient trials with valid recordings (5 or less recordings; Germany: *n* = 4; Japan: *n* = 7), and a child that did not say anything in this task (Cameroon: *n* = 1).

#### Eye-tracking task

Children saw 40 real, semantic pictures which displayed objects in front of a simple background [6, 11], see Fig 3A. These pictures were similar to those of the picture description tasks and taken from the same sources. Furthermore, 40 abstract, non-semantic pictures with abstract objects in front of abstract backgrounds were shown. We used artificial objects from experimental psychology (greebles, fribbles, geons and multipart geons, e.g., [31], taken from an online database: http://wiki.cnbc.cmu.edu/Novel_Objects) and fractal pictures (20 pictures; created with quadrium 2.0, quadrium.en.softonic.com) or details of abstract drawings (20 pictures) as backgrounds, cf. [32]. The pictures of both sets were presented in a randomized order.

Trials started with a fixation dot (shown for 1 s), followed by the stimulus (5 s). The instruction for the children was to “…look at the pictures attentively…”, while the experimenter sat beside them.

Participants’ gaze behavior was recorded binocularly, at a sampling rate of 60 Hz or higher. In Cameroon and Germany we used a SMI redm250 (SensoMotoric Instruments GmbH, Teltow, Germany), in Japan we used a Tobii X60 (Tobii Technology, Stockholm, Sweden) eye-tracking system. Individual fixations were identified by the respective eye-tracking software (Cameroon and Germany: BeGaze, Version 3.5.101; Japan: Tobii Studio, Version 3.3.2). Fixations were then exported for further analyses in MATLAB (Version 2013a). Areas of interest (AOI) were drawn around the objects of each stimulus using BeGaze (Version 3.5.101) and imported into MATLAB as a template, to assure that AOIs were identical across contexts.

To quantify participants’ visual attention to the object, relative to the background, we calculated an object focus score: The duration of all fixations made into the AOI of the object was divided by the duration of all fixations on the picture within the 5 s of stimulus presentation. Thus, a score of 1 would indicate that the participant only looks at the object, whereas a score of 0 would indicate that a participant does only look at the background. As a further measure for participants’ object bias, we looked at the average latency, until the first fixation entered the AOI of the object (cf. [6]).

Data from fourteen children were excluded from this task, due to a lack of motivation to participate in both conditions (Germany: *n* = 1; Japan: *n* = 4), insufficient trials with valid recordings per condition (i.e., less than 10 trials with more than 1.5 seconds or 5 fixation with recorded gaze behavior, which indicates that children did not attend the stimulus or where staring; Germany: *n* = 2; Cameroon: *n* = 1; Japan: *n* = 3), inconsistent gaze behavior (less than 15% of presentation time used for object exploration in one condition, indicating bad eye-tracking recordings; Germany: *n* = 2), and technical problems during assessment (Japan: *n* = 1).

#### Parallel action task

In the parallel action task children saw a video with two action sequences shown in parallel (i.e., the display was split in two halves), see Fig 1D. Specifically, each video displayed a pair of hands producing artificial handicrafts from four different colored pellets of modelling clay, two additional materials (e.g., wires or sticks), and two tools (e.g., a pounder or a scarper). In both action sequences, an artificial object was produced in nine action steps. Specifically, each pellet was modified, with a tool or with the hands, before all pellets were assembled, including the two additional materials. The display of the action sequences was precisely timed, such that each action step took 20 seconds, the hands in both sequences started simultaneously and ended each action step simultaneously in their starting position, at the lower edge of the screen. The side of the presentation of the two action sequences was counterbalanced across participants.

Children were not told that they would have to reproduce the shown actions, but instructed to watch exactly, what is done in the videos. During the presentation of the video, the gaze behavior was recorded, using the same apparatus as in the eye-tracking task, see above. After the presentation of the video, children were given two trays with the pellets, materials and tools from the presented action sequences, one after another, and were asked to reproduce what they had seen in the video. For each tray children had seven minutes of time, or until the child indicated that they had finished. The experimenter sat beside the child, pretending to read, but gave a series of standardized cues (after one and two minutes: “…Try to do exactly what was done in the video.”; after four minutes the experimenter indicated the unused pellets, materials and tools: “Do you remember what was done with […], just do what you think was done”; after 6 minutes: “You have to finish soon…”). After seven minutes the tray was taken away and the second tray was handed to the child for another seven minutes. Because pilot data indicated that one of the action sequences was somewhat more difficult to reproduce, all children started with the more difficult object, before they received the tray with the somewhat easier object. This was to avoid stark differences in the number of actions reproduced, and our interest to look at sequences that were displayed in parallel.

The analysis focused on, first, children’s gaze behavior when watching the action sequences and, second, their reproduced action steps. After preprocessing the eye-tracking data as in the scene exploration task (see above), we calculated the number of saccades that shifted from one action sequence to the other (e.g., from left to right or vice versa), divided by the overall gaze time in seconds. Furthermore, to estimate the distribution of their attention to both sequences, we computed the proportion of time that children focused on the preferred action sequence (i.e., the action sequence that the child looked at for longer). That is, a score of 1 would indicate that the child only attended to one of the sequences, whereas a score of 0.6 would indicate that the child looked for 60% of the time to the preferred sequence and only 40% of the time to the non-preferred sequence. With regard to children’s ability to reproduce actions of both sequences, we looked at correctly reproduced actions, namely correct tool/material-pellet combinations (e.g., pounder to red pellet), followed by the correct action (e.g., squeezing the red pellet). We looked at the proportion of reproduced actions that were shown in parallel. This is, we divided the number correctly reproduced actions, which were shown in parallel, by the total number of actions that where reproduced. Inter-rater agreements for correctly reproduced actions were assessed for > 20% of the data and Cohen’s kappa for each context was κ > .81.

Nine children were excluded from the behavioral analyses of this task, due to a lack of motivation to continue with the task or the data assessment (Germany: *n* = 3; Japan: *n* = 4), and a technical error occurring during the assessment (Germany: *n* = 1). Eight additional participants were excluded only from the eye-tracking analyses due to incomplete or insufficient eye-tracking recordings (Germany: *n* = 4; Japan: *n* = 4)

#### Rule-based game

In the rule-based game children where shown a video with two people playing a social game (Fig 4A). Each turn of the game comprised taking a card with a certain pattern (e.g., a square), sorting the pattern into an envelope on a cardboard with a certain shape (e.g., into a star shape) and to perform a certain action (e.g., clapping hands). Each of four moves was presented twice in the video. The stimulus videos were videotaped for each context with local research assistants. The timing (20 s per move) and the speech was fully scripted. In particular, the actors labeled what they were doing (“these are stars […], I do clap, clap, clap” ) but did not mention the rules explicitly (e.g., combination of pattern on the card and shape of the envelope).

Before the game started, children were instructed to watch carefully and explicitly asked to remember the rules and the actions, because they would play the game with the experimenter later on. After the presentation of the video, the child and the experimenter moved to a table and played the rule-action game. The child started and the cards were sorted in a way that the child drew each pattern first, before the experimenter drew the same pattern. It was then observed, whether the child remembered the rule (pattern on card to shape of envelope allocation) and whether they performed an action that was demonstrated in the video. If the child hesitated and could not remember a rule or an action, the experimenter asked: “Think about it, do you remember where this card was placed/which action was done next. Otherwise you may guess and try.”

Inter-rater agreements for the comprehension of rules and correctly reproduced actions were assessed for > 20% of the data (Cohen’s kappas for each context was κ_rule comprehension_ = 1.0, and κ_action reproduction_ > .91).

Two children were excluded from the behavioral analyses of this task, due to a lack of motivation to continue the data assessment (Germany: *n* = 1), and a procedural error by the experimenter (Germany: *n* = 1).

## Supporting information

S1 File Data file. (SAV)

S2 File Illustration of the video of the parallel action task (cut). (AVI)
